# Supplementary material for: Translation, cultural adaptation, and validation of the Geriatric Depression Scale – Short Form into Bengali among elderly people in Bangladesh
Source: PLOS Glob Public Health. 2026 Mar 19;6(3):e0005341. doi: 10.1371/journal.pgph.0005341 (PMC13001932; doi:10.1371/journal.pgph.0005341)
Supplement: Attachment — Submitted filename: PGPH-D-25-00588_reviewerkrl.docx [file pgph.0005341.s002.docx]

PLOS Global Public Health

Translation, Cultural Adaptation, and Validation of the Geriatric Depression Scale – Short Form into Bengali among Bangladeshi Elderly People

--Manuscript Draft--

| Manuscript Number: | PGPH-D-25-00588 |
| --- | --- |
| Article Type: | Research Article |
| Full Title: | Translation, Cultural Adaptation, and Validation of the Geriatric Depression Scale – Short Form into Bengali among Bangladeshi Elderly People |
| Short Title: | Translation and Validation- Geriatric Depression Scale–Short Form, Bengali, Bangladeshi |
| Corresponding Author: | Mahmud Hassan Arif, MRCP, FCPS, SCE (Geriatric Medicine) Chittagong Medical College  Chittagong, BANGLADESH |
| Order of Authors: | Mahmud Hassan Arif, MRCP, FCPS, SCE (Geriatric Medicine) |
|  | Panchanan Acharjee |
|  | Mohammad Waliul Hasnat Sajib |
|  | Md. Maruful Haque |
|  | Fahmida Hoque Rimti |
|  | Mohiuddin Ahsanul Kabir Chowdhury |
| Keywords: |  |
| Abstract: | Introduction  Depression is a major health concern among the elderly, affecting their quality of life. The Geriatric Depression Scale – Short Form (GDS-15) is commonly used to screen for depression in older adults. This study aimed to translate, culturally adapt, and validate the GDS-15 into Bengali for use among elderly people in Bangladesh.  Methods  The GDS-15 was translated into Bengali, followed by cultural adaptation. A cross- sectional study was conducted with 100 elderly participants aged 60 years, and above in Chittagong Medical College Hospital. Psychometric validation was performed using reliability testing, chi-square analysis, ANOVA, and principal component analysis (PCA).  Results  The Bengali version of the GDS-15 demonstrated strong construct validity, with significant correlations between the original and translated versions of the items (r = 0.9788, p < 0.001). However, reliability analysis showed a low Cronbach's alpha (0.4753), indicating suboptimal internal consistency. The PCA revealed multiple components, suggesting the multidimensionality of the scale in this population.  Conclusion  The Bengali version of the GDS-15 is valid but requires further refinement to improve its reliability. Cultural adaptation ensured the tool’s appropriateness while additional modifications may enhance its internal consistency. The Bengali GDS-15 has the potential to serve as a useful screening tool for geriatric depression in Bangladesh with further improvements. |
| Additional Information: |  |
| Question | Response |

| Financial Disclosure  Enter a financial disclosure statement that describes the sources of funding for the work included in this submission and the role the funder(s) played. This includes grants and any commercial funding of the work or authors.  This statement will be typeset if the manuscript is accepted for publication.  Please review the [submission guidelines](http://journals.plos.org/globalpublichealth/s/submission-guidelines#loc-financial-disclosure-statement) and the instructions link below for detailed requirements and guidance. | none |
| --- | --- |
| Competing Interests  On behalf of all authors, disclose any competing interests that could be perceived to bias this work.  This statement will be typeset if the manuscript is accepted for publication.  Please review the instructions link below and PLOS Global Public Health’s [competing interests](https://journals.plos.org/globalpublichealth/s/competing-interests) policy to determine what information must be disclosed at submission. | no conflict of interest |
| Data Availability  Before publication, Authors are required to make fully available and without restriction all data underlying their findings. Please see our [PLOS Data](https://journals.plos.org/globalpublichealth/s/data-availability) [Policy](https://journals.plos.org/globalpublichealth/s/data-availability) page for detailed information on this policy.  A Data Availability Statement, detailing where the data can be accessed, is required at first submission. Insert your | available on request |

| Data Availability Statement in the box below.  Please see the [data reporting](https://journals.plos.org/globalpublichealth/s/submission-guidelines#loc-data-reporting) section of our submission guidelines for instructions on what you need to include in your Data Availability Statement.  This statement will be typeset if the manuscript is accepted for publication.  PLOS allows rare exemptions to address legal and ethical concerns. If you have legal or ethical restrictions, please use the box below to detail these in full sentences for the Journal team to consider. |  |
| --- | --- |

Manuscript	[Click here to access/download;Manuscript;GDS_Paper_Main](https://www2.cloud.editorialmanager.com/pgph/download.aspx?id=518227&guid=0df47524-8eed-46ba-86ba-2ef8646049fd&scheme=1)[<https://www2.cloud.editorialmanager.com/pgph/download.aspx?id=518227&guid=0df47524-8eed-46ba-86ba-2ef8646049fd&scheme=1> Arif - Corxn 250325.docx](https://www2.cloud.editorialmanager.com/pgph/download.aspx?id=518227&guid=0df47524-8eed-46ba-86ba-2ef8646049fd&scheme=1)

Translation, Cultural Adaptation, and Validation of the Geriatric Depression Scale – Short Form into Bengali among Bangladeshi Elderly People: a hospital-based study

Mahmud Hassan Arif,^1*^Panchanan Acharjee,^1^Mohammad Waliul Hasnat Sajib,^2^Md. Maruful Haque,^3^Fahmida Hoque Rimti,^1^ Mohiuddin Ahsanul Kabir Chowdhury^4^

^1^ Chittagong Medical College, Chittagong, Bangladesh

^2^Tangail Medical College, Tangail, Bangladesh

^3^Shaheed Ziaur Rahman Medical College,Bogura, Bangladesh

^4^Asian University for Women, Chittagong, Bangladesh

^*^ Corresponding author Mahmud Hassan Arif [arif.mahmud@gmail.com](mailto:arif.mahmud@gmail.com)

# Abstract:

Introduction: Depression is a major health concern among the elderly, affecting their quality

of life. The Geriatric Depression Scale – Short Form (GDS-15) is commonly used to screen for depression in older adults. This study aimed to translate, culturally adapt, and validate the GDS-15 into Bengali for use among elderly people in Bangladesh.

Methods: The GDS-15 was translated into Bengali, followed by cultural adaptation. A cross-

sectional study was conducted with 100 elderly participants aged 60 years, and above in Chittagong Medical College Hospital. Psychometric validation was performed using reliability testing, chi-square analysis, ANOVA, and principal component analysis (PCA).

Results: The Bengali version of the GDS-15 demonstrated strong construct validity, with

significant correlations between the original and translated versions of the items (r = 0.9788, p < 0.001). However, reliability analysis showed a low Cronbach's alpha (0.4753), indicating suboptimal internal consistency. The PCA revealed multiple components, suggesting the multidimensionality of the scale in this population.

Conclusion: The Bengali version of the GDS-15 is valid but requires further refinement to

improve its reliability. Cultural adaptation ensured the tool’s appropriateness while additional modifications may enhance its internal consistency. The Bengali GDS-15 has the potential to serve as a useful screening tool for geriatric depression in Bangladesh with further improvements.

# Introduction:

Depression is a common mental health condition among elderly individuals, significantly impacting their quality of life, physical health, and overall well-being.(1) The prevalence of geriatric depression is particularly concerning in low- and middle-income countries, where the elderly population often faces unique challenges, such as limited access to healthcare, social isolation, and increased economic dependency.(2) In Bangladesh, these challenges are further exacerbated by cultural stigmatization of mental health issues, leading to underdiagnosis and undertreatment of depression among older adults.(3) Early detection of depressive symptoms is therefore crucial for improving the mental health and quality of life of elderly people.(4)

The Geriatric Depression Scale (GDS) is one of the most widely used screening tools for detecting depression in elderly populations. (5, 7) The Short Form of the GDS (GDS-15) is a concise version comprising 15 items, designed to facilitate rapid assessment in clinical and community settings. (6, 8) Its brevity and ease of use make it particularly suitable for populations with low literacy and limited access to healthcare professionals, such as those in rural Bangladesh.(9) However, before the GDS-15 can be used effectively in a new cultural setting, it is essential to ensure that the tool is linguistically and culturally appropriate for the target population.(10)

Translation and cultural adaptation are necessary to maintain the original tool's conceptual and functional equivalence in a different language and cultural context.(11) These processes help to ensure that the translated items accurately reflect the intended meanings and that the tool is appropriate for the cultural norms and experiences of the target population.(11) In addition, psychometric validation is needed to assess the reliability and validity of the

translated version, ensuring that it measures depression accurately and consistently among the target population.(12)

This study aimed to validate the GDS-15 for use among elderly individuals in Bangladesh which seeks to provide a valid and reliable screening tool for geriatric depression in the country, contributing to improved mental health care for the country's aging population.

# Materials and Methods

*Study Design and Study setting*

We conducted a cross-sectional study involving 98 participants aged 60 years or older? Who were seen at the Chittagong Medical College Hospital for x/y/z admission/visit reasons.

*Translation and Cultural Adaptation*

The GDS-15 was translated into Bengali following a standard process involving forward translation, backward translation, and a review by an expert committee comprising bilingual professionals. Cultural adaptation was conducted to ensure the scale was relevant to the context of Bangladeshi elderly individuals, including modifications to language and cultural expressions.

*Data Collection*

The translated version of the GDS-15 was administered by trained interviewers. In addition to the GDS-15, socio-demographic data including age, sex, education level, and other relevant characteristics were collected.

*Sample size and sampling technique*

The study included a total of 98 elderly participants selected through purposive sampling to explore the socio-demographic, clinical, and lifestyle factors associated with depression in this population. Participants were aged 60 years or older and were recruited from diverse community settings to ensure variability in socio-demographic and health-related characteristics. The sample size was determined to provide sufficient statistical power to detect meaningful associations across depression categories (no depression, mild depression, and moderate depression). Efforts were made to include individuals with varying levels of education, marital status, family types, and comorbid conditions to ensure the generalizability

of findings within the elderly population in the study setting. This targeted approach facilitated the identification of key factors influencing depression in older adults.

*Statistical Analysis*

Data was analyzed using Stata BE18.0. Descriptive statistics were calculated for the socio- demographic characteristics of the participants. The reliability of the scale was assessed using Cronbach's alpha. Chi-square tests, correlation analyses, ANOVA, and principal component analysis (PCA) were conducted to validate the structure and consistency of the Bengali version of the GDS-15.

*Ethical Approval*

The ethical approval was obtained from the Institutional Review Board of the Chittagong Medical College Hospital. (Memo no: CMC/PG/2022/248)

# Results

## Socio-demographic Characteristics

The study evaluated socio-demographic characteristics across three depression categories: no depression, mild depression, and moderate depression. Among ninety-eight study participants, 61.2% were diagnosed with mild depression and 34.7% were diagnosed with moderate depression. The mean age was 66.6 ± 4.4 years, with no statistically significant differences among the depression categories (p = 0.439). The proportion of males (77.5%) was higher than females (22.5%) across all groups, and sex differences were not statistically significant (p = 0.462). Most participants had completed 11–12 years or ≥13 years of education, and there was no significant association with depression severity (p = 0.667). Most participants lived in joint families (80.6%) and were married (77.6%). Religion, smoking status, and psychiatric or family history of psychiatric disorders showed no significant

association with depression severity. The socio-demographic characteristics are detailed out in Table 1.

Table 1: Socio-demographic characteristics of the participants

| **Characteristics** | **No Depression** | **Mild Depression** | **Moderate Depression** | **Total** | **P-**  **Value** |
| --- | --- | --- | --- | --- | --- |
| Age, in years (Mean ± SD) | 64.3 ± 3.0 | 67.2 ± 4.2 | 65.8 ± 4.8 | 66.6 ± 4.4 | 0.439 |
| 60-64 years | 1 (25.0) | 20 (33.0) | 17 (50.0) | 38 (38.8) |  |
| 65-69 years | 3 (75.0) | 20 (33.3) | 10 (29.4) | 33 (33.7) | 0.360 |
| 70-74 years | 0 | 16 (26.7) | 5 (14.7) | 21 (21.4) |  |
| More than 75 years | 0 | 4 (6.7) | 2 (5.9) | 6 (6.1) |  |

Sex

Male	3 (75.0)	49 (81.7)	24 (31.6)	76 (77.5)	0.462

Female	1 (25.0)	11 (18.3)	10 (29.4)	22 (22.5)

Education

| 6-10 years | 0 (0.0) | 3 (5.0) | 1 (2.9) | 4 (4.1) | 0.667 |
| --- | --- | --- | --- | --- | --- |
| 11-12 years | 1 (25.0) | 27 (45.0) | 19 (55.9) | 47 (47.9) |  |
| ≥ 13 years | 3 (75.0) | 30 (50.0) | 14 (41.2) | 47 (47.9) |  |
| Family type |  |  |  |  |  |
| Nuclear | 1 (25.0) | 10 (16.7) | 4 (11.8) | 15 (15.3) | 0.672 |
| Joint | 3 (75.0) | 46 (76.7) | 30 (88.3) | 79 (80.6) |  |
| Extended | 0 (0.0) | 4 (6.7) | 0 (0.0) | 4 (4.1) |  |
| Marital status |  |  |  |  | 0.708 |

| Married | 3 (75.0) | 45 (75.0) | 28 (82.4) | 76 (77.6) |
| --- | --- | --- | --- | --- |
| Widowed /Widower | 1 (25.0) | 15 (25.0) | 6 (17.7) | 22 (22.5) |
| Religion |  |  |  |  |
| Muslim | 3 (75.0) | 48 (80.0) | 30 (88.2) | 81 (82.7) 0.802 |
| Non-Muslim | 1 (25.0) | 12 (20.0) | 4 (11.8) | 17 (17.3) |

## Lifestyle and health-related characteristics

There were no significant associations between smoking status and depression severity (p=0.673). Among participants with mild depression, 61.7% were ex- or current smokers, while 38.3% were non-smokers. Similarly, 52.9% of participants with moderate depression were ex- or current smokers. The presence of comorbid conditions was prevalent across all groups, with 96.9% of the total sample reporting at least one comorbidity (p=0.375). Additionally, a family history of psychiatric disorders was uncommon, with only 6.1% of participants reporting such a history and no significant differences observed between depression categories (p=0.863). Past psychiatric history was similarly low, with 5.1% of participants reporting a prior diagnosis, and this was not significantly associated with depression severity (p=0.655). These findings suggest that while comorbid conditions are highly prevalent among the elderly, other factors may play a more significant role in the development and severity of depressive symptoms. The details are provided in Table 2.

Table 2: Lifestyle and Health-related characteristics (N=98)

**Characteristics**

**No Depression**

**Mild Depression**

**Moderate Depression**

**Total**

**P-**

**Value**

Smoking status

| Non-smoker | 2 (50.0) | 23 (38.3) | 16 (47.1) | 41 (41.8) | 0.673 |
| --- | --- | --- | --- | --- | --- |
| Ex- or Current Smoker | 2 (50.0) | 37 (61.7) | 18 (52.9) | 57 (58.2) |  |
| Comorbid conditions |  |  |  |  |  |
| No | 0 (0.0) | 3 (5.0) | 0 (0.0) | 3 (3.1) | 0.375 |
| Yes | 4 (100.0) | 57 (95.0) | 34 (100.0) | 95 (96.9) |  |

Family History of Psychiatric Disorder

No	4 (100.0)	56 (93.3)	32 (94.1)	92 (93.8)	0.863

Yes	0 (0.0)	4 (6.7)	2 (5.9)	6 (6.1)

Past History of Psychiatric Disorder

No	4 (100.0)	56 (93.3)	33 (97.1)	93 (94.9)	0.655

Yes	0 (0.0)	4 (6.7)	1 (2.9)	5 (5.1)

## Internal Consistency

The reliability analysis using Cronbach's alpha for the 15-item GDS-15 scale revealed a coefficient of 0.5638, which indicates low internal consistency. The Bengali translated GDS- 15 scale yielded a Cronbach’s alpha of 0.5408.

## Bivariate Analysis

The bivariate analysis compared the agreement between the English and Bengali versions of GDS-15. High agreement was observed for all items, with percentages ranging from 84.6% to 98.4% (p < 0.001 for all).(Table 3)

Table 3: Comparison between English and Bengali version of GDS scale

| **GDS GDS P-**  **Questions Agreement**  **English Translated value** | | | | |
| --- | --- | --- | --- | --- |
| Are you basically satisfied with your life? | 60 (61.2) | 61 (62.6) | 98.4% | <0.001 |
| Have you dropped many of your activities | 75 (76.5) | 75 (76.5) | 97.3% | <0.001 |
| and interests? |  |  |  |  |
| Do you feel that your life is empty? | 32 (32.7) | 33 (33.7) | 97.0% | <0.001 |
| Do you often get bored? | 41 (41.4) | 42 (42.9) | 97.6% | <0.001 |
| Are you in good spirits most of the time? | 45 (45.9) | 46 (46.9) | 97.8% | <0.001 |
| Are you afraid that something bad is going to | 57 (58.2) | 56 (57.1) | 98.2% | <0.001 |
| happen to you? |  |  |  |  |
| Do you feel happy most of the time? | 55 (56.1) | 60 (61.2) | 90.0% | <0.001 |
| Do you often feel helpless? | 59 (60.2) | 58 (59.2) | 98.3% | <0.001 |
| Do you prefer to stay at home, rather than | 65 (66.3) | 68 (69.4) | 94.1% | <0.001 |
| going out and doing new things? |  |  |  |  |
| Do you feel you have more problems with | 53 (54.1) | 56 (57.1) | 87.5% | <0.001 |
| memory than most people? |  |  |  |  |
| Do you think it is wonderful to be alive? | 55 (56.1) | 60 (61.2) | 88.3% | <0.001 |
| Do you feel pretty worthless the way you are | 46 (46.9) | 52 (53.1) | 84.6% | <0.001 |
| now? |  |  |  |  |
| Do you feel full of energy? | 37 (37.8) | 39 (39.8) | 92.3% | <0.001 |
| Do you feel that your situation is hopeless? | 52 (53.1) | 54 (55.1) | 92.3% | <0.001 |
| Do you think that most people are better off | 42 (42.9) | 46 (46.9) | 89.1% | <0.001 |
| than you are? |  |  |  |  |

## Diagnostic Performance

Table 4 shows the diagnostic accuracy of the translated scale. The GDS-15 scale demonstrated good diagnostic accuracy. Sensitivity for detecting mild depression was 100%, and specificity was 75%, with an ROC area of 87.5% (63.0–100.0%). (Figure 1) For moderate/severe depression, sensitivity was 94.1%, specificity was 84.4%, and the ROC area was 89.3% (83.3–95.3%). (Figure 2)

Table 4: Sensitivity and Specificity of Translated GDS scale

| **Diagnosis** | **Sensitivity** | **Specificity** | **ROC area** |
| --- | --- | --- | --- |
| Mild Depression | 100% | 75% | 87.5 (63.0 – 100.0)% |
| Moderate / Severe Depression | 94.1% | 84.4% | 89.3 (83.3-95.3)% |

Figure 1: ROC Curve for mild depression

Figure 2: ROC Curve for moderate/severe depression

## Factor Analysis

Principal component analysis (PCA) revealed that five components had eigenvalues greater than 1, explaining 52.47% of the cumulative variance. The first component explained 15.3%, suggesting that the scale captures multiple dimensions of depression. Variance proportions were similar between the English and Bengali versions, indicating comparable dimensionality. (Table 5)

Table 5: Principal Component Analysis of the GDS scale

| **Questions** | **GDS English GDS Translated** |
| --- | --- |
|  | **Proportion Proportion**  **Eigenvalue Eigenvalue**  **(%) (%)** |

| Are you basically satisfied with your life? | 2.52 | 16.8 | 2.45 | 16.3 |
| --- | --- | --- | --- | --- |
| Have you dropped many of your activities | 1.59 | 10.6 | 1.71 | 11.4 |
| and interests? |  |  |  |  |
| Do you feel that your life is empty? | 1.50 | 10.0 | 1.47 | 9.8 |
| Do you often get bored? | 1.37 | 9.2 | 1.43 | 9.6 |
| Are you in good spirits most of the time? | 1.18 | 7.9 | 1.05 | 7.0 |
| Are you afraid that something bad is | 1.05 | 7.0 | 0.99 | 6.6 |
| going to happen to you? |  |  |  |  |
| Do you feel happy most of the time? | 0.95 | 6.4 | 0.95 | 6.3 |
| Do you often feel helpless? | 0.89 | 5.9 | 0.89 | 6.0 |
| Do you prefer to stay at home, rather than | 0.78 | 5.2 | 0.82 | 5.5 |
| going out and doing new things? |  |  |  |  |
| Do you feel you have more problems with | 0.69 | 4.6 | 0.72 | 4.8 |
| memory than most people? |  |  |  |  |
| Do you think it is wonderful to be alive? | 0.62 | 4.1 | 0.67 | 4.5 |
| Do you feel pretty worthless the way you | 0.59 | 3.9 | 0.59 | 3.9 |
| are now? |  |  |  |  |
| Do you feel full of energy? | 0.49 | 3.3 | 0.49 | 3.3 |
| Do you feel that your situation is | 0.41 | 2.7 | 0.44 | 2.9 |
| hopeless? |  |  |  |  |
| Do you think that most people are better | 0.37 | 2.4 | 0.33 | 2.2 |

off than you are?

## Cultural Adaptation

Cultural adaptation ensured that the Bengali-translated GDS-15 reflected local expressions and culturally relevant concepts, enhancing its applicability to the elderly population in Bangladesh. An expert committee reviewed and supported the comprehensibility and cultural appropriateness of the translated items.

# Discussion

The present study aimed to evaluate the reliability, validity, and cultural adaptation of the Geriatric Depression Scale-15 (GDS-15) for use among elderly individuals in Bangladesh. The validation of the Bengali version of the GDS-15 in this study contributes to the growing body of evidence on adapting geriatric depression screening tools for culturally diverse populations. While the tool showed high diagnostic accuracy and strong agreement between the original and translated versions, the low internal consistency suggests challenges common to similar cross-cultural adaptations. For instance, studies in non-Western settings, such as the Igbo adaptation by Mgbeojedo et al. (2022) and Sultana et al. (2022) in Bangladesh, similarly reported varying levels of reliability due to cultural nuances and differing interpretations of specific items.(9, 10)

The socio-demographic analysis revealed no significant associations between depression severity and variables such as age, sex, education, marital status, or family structure. This suggests that depression among the elderly in this population is likely influenced by other factors, such as social isolation, chronic illness, or cultural perceptions of mental health, which were not directly assessed in this study.(13-15)However, there are a few studies that documented the association of geriatric depression with socio-demographic factors. For

instance, a survey conducted among the Thai elderly population found an association of depression with old age, being single, drinking alcohol daily, and having diabetes.(16) Another study showed that marital status is somehow linked to geriatric depression.(17) In Bangladesh, a study documented similar results where being without a spouse was identified as a risk factor.(18)A study looked into the risk factors for depression among the elderly population in Bangladesh and found similar results to this study.(19)

The low Cronbach's alpha values (0.5638 for the English version and 0.5408 for the Bengali version) highlight the need for scale refinement, a limitation noted in comparable research. While these values fall below the commonly accepted threshold of 0.7, they are equivalent to other studies evaluating the GDS in culturally diverse settings. This result may indicate that some items on the scale are less relevant or less consistently understood in the Bangladeshi context. For example, questions reflecting specific cultural or social norms might contribute to lower reliability. In Krishnamoorthy et al.’s meta-analysis (2020), the reliability of the GDS-15 varied across cultures, with adaptations often requiring modifications to address local norms.(7)Refinement of the scale, such as revising or removing items with low item- total correlations, may improve its psychometric properties.

Despite low internal consistency, the translated GDS-15 demonstrated strong agreement with the original English version, with all items showing high concordance (≥84.6%). This suggests that the translation process successfully preserved the content and intent of the original scale. Furthermore, the tool demonstrated excellent diagnostic performance, with high sensitivity and specificity for detecting mild and moderate/severe depression. The ROC areas (87.5% for mild and 89.3% for moderate/severe depression) underscore the effectiveness of the GDS-15 as a screening tool in this population. The high sensitivity and specificity of the Bengali GDS-15 (87.5% for mild depression and 89.3% for moderate/severe depression) are consistent with other regional adaptations, such as those observed in Sultana

et al.’s ( insert year) study in Bangladesh.(9) This underscores the utility of the GDS-15 as a screening tool in low-resource settings where mental health services are limited. However, the identified multidimensionality suggests that a more tailored approach may be needed to fully capture the construct of depression in the Bangladeshi elderly population. These findings are consistent with previous studies that validated the GDS-15 in other non-Western settings.

The factor analysis revealed that depression in this population is multidimensional, with five components explaining 52.47% of the total variance. The first component accounted for only 15.3% of the variance, suggesting that the scale does not capture a single dominant factor of depression. This is an important finding, as it indicates that the construct of depression among elderly individuals in Bangladesh may differ from that in Western populations, where the GDS was originally developed. The identified dimensions may reflect unique cultural and social determinants of mental health in this setting, warranting further qualitative research.

This finding emphasizes the multidimensional nature of depression in non-Western contexts, as indicated by the principal component analysis in this study, which identified five distinct factors accounting for 52.47% of the variance. Such multidimensionality aligns with findings from Shin et al. (2019), who observed variations in depressive symptomatology influenced by social and cultural factors.(8)

The cultural adaptation process was instrumental in ensuring the scale’s comprehensibility and relevance to the elderly Bangladeshi population. Modifications to language and phrasing were guided by an expert committee, incorporating cultural nuances and local expressions. This adaptation likely contributed to the high agreement between the original and translated versions. However, the lower internal consistency highlights the need for further adjustments to improve the scale’s reliability without compromising its cultural appropriateness.

# Strengths and Limitations

A key strength of this study was the rigorous translation and cultural adaptation process, which ensured that the Bengali GDS-15 reflected local linguistic and cultural expressions. Similar methodologies have been advocated by Borsa et al. (2012) and Arafat et al. (2016), who stressed the importance of preserving conceptual equivalence in cross-cultural validations.(11, 12) Additionally, the inclusion of a diverse elderly population enhances the generalizability of the findings within Bangladesh.

Despite these efforts, some items may have been less culturally relevant, potentially contributing to lower internal consistency. Future revisions should consider qualitative methods, such as focus groups, to explore cultural perceptions of the scale’s items. Several other limitations should be noted. First, the sample size, while adequate for the analyses conducted, may not capture the full heterogeneity of the elderly population. Second, the study relied on self-reported data, which may be subject to social desirability bias, particularly in a cultural context where mental health remains stigmatized. Finally, the low internal consistency of the GDS-15 indicates that additional refinement of the scale is necessary for optimal use in this setting.

# Future Directions

Future research should explore qualitative approaches to better understand the dimensions of depression among elderly individuals in Bangladesh. Focus group discussions or in-depth interviews could help identify culturally specific factors influencing depressive symptoms, which may inform the development of a more tailored assessment tool. Additionally, longitudinal studies are needed to examine the predictive validity of the GDS-15 and its utility in tracking changes in depressive symptoms over time.

# Conclusion

In conclusion, the GDS-15 demonstrates promise as a screening tool for depression among elderly individuals in Bangladesh. While the translation and cultural adaptation process ensured high agreement and diagnostic performance, the low internal consistency underscores the need for further refinement of the scale. Addressing these limitations will enhance the utility of the GDS-15 in identifying and addressing mental health needs in this vulnerable population, contributing to improved geriatric care and mental health outcomes in Bangladesh.

Acknowledgements

We want to acknowledge the authority of Chittagong Medical College Hospital to allow us to conduct the study in the hospital. We are also grateful to the caregivers of the elderly patients admitted to the hospital. Furthermore, the contribution of the geriatric population in this paper is invaluable.

# References:

1. Sivertsen H, Bjørkløf GH, Engedal K, Selbæk G, Helvik A-S. Depression and quality of life in older persons: a review. Dementia and geriatric cognitive disorders. 2015;40(5- 6):311-39.
2. Brinda EM, Rajkumar AP, Attermann J, Gerdtham UG, Enemark U, Jacob KS. Health, social, and economic variables associated with depression among older people in low and middle income countries: World Health Organization study on global AGEing and adult health. The American Journal of Geriatric Psychiatry. 2016;24(12):1196-208.
3. Faruk MO, Rosenbaum S. Mental illness stigma among indigenous communities in Bangladesh: a cross-sectional study. BMC psychology. 2023;11(1):216.
4. Faruk MO, Khan AH, Chowdhury KUA, Jahan S, Sarker DC, Colucci E, et al. Mental illness stigma in Bangladesh: Findings from a cross-sectional survey. Cambridge Prisms: Global Mental Health. 2023;10:e59.
5. Yesavage JA, Brink TL, Rose TL, Lum O, Huang V, Adey MB, Leirer VO: Development and validation of a geriatric depression screening scale: A preliminary report. Journal of Psychiatric Research 17: 37-49, 1983.
6. Sheikh JI, Yesavage JA: Geriatric Depression Scale (GDS): Recent evidence and development of a shorter version. Clinical Gerontology : A Guide to Assessment and Intervention 165-173, NY: The Haworth Press, 1986.
7. Krishnamoorthy Y, Rajaa S, Rehman T. Diagnostic accuracy of various forms of geriatric depression scale for screening of depression among older adults: Systematic review and meta-analysis. Archives of gerontology and geriatrics. 2020;87:104002.
8. Shin C, Park MH, Lee S-H, Ko Y-H, Kim Y-K, Han K-M, et al. Usefulness of the 15- item geriatric depression scale (GDS-15) for classifying minor and major depressive disorders among community-dwelling elders. Journal of affective disorders. 2019;259:370-5.
9. Sultana N, Nguyen TT, Hossain A, Asaduzzaman M, Nguyen MH, Jahan I, et al.

Psychometric Properties of the short-form Geriatric Depression Scale (GDS-SF) and its associated factors among the elderly in Bangladesh. International journal of environmental research and public health. 2022;19(13):7935.

10. Mgbeojedo UG, Akosile CO, Ezugwu JC, Okoye EC, John JN, Ani KU, et al. Cross- cultural adaptation and validation of the 15-item Geriatric Depression Scale (GDS-15) into Igbo language: a validation study. Health and quality of life outcomes. 2022;20(1):22.
11. Borsa JC, Damásio BF, Bandeira DR. Cross-cultural adaptation and validation of psychological instruments: Some considerations. Paidéia (Ribeirão Preto). 2012;22:423-32.
12. Arafat S, Chowdhury HR, Qusar M, Hafez M. Cross cultural adaptation and psychometric validation of research instruments: a methodological review. Journal of Behavioral Health. 2016;5(3):129-36.
13. Tomaka J, Thompson S, Palacios R. The relation of social isolation, loneliness, and social support to disease outcomes among the elderly. Journal of aging and health. 2006;18(3):359-84.
14. Conner KO, Copeland VC, Grote NK, Koeske G, Rosen D, Reynolds III CF, et al.

Mental health treatment seeking among older adults with depression: the impact of stigma and race. The American Journal of Geriatric Psychiatry. 2010;18(6):531-43.

15. Bhut KN. The Impact of Social isolation and loneliness on mental health and overall wellbeing of geriatric Population: A systemic literature review. PQDT-Global. 2024.
16. Aung TNN, Moolphate S, Koyanagi Y, Angkurawaranon C, Supakankunti S, Yuasa M, et al. Depression and associated factors among community-dwelling Thai older adults in northern Thailand: the relationship between history of fall and geriatric depression. International journal of environmental research and public health. 2022;19(17):10574.
17. Taqui AM, Itrat A, Qidwai W, Qadri Z. Depression in the elderly: Does family system play a role? A cross-sectional study. BMC psychiatry. 2007;7:1-12.
18. Hossain MK, Islam MN, Uddin MT, Hossain MS. Understanding the impact of socioeconomic and health factors on geriatric depression: A comparative study in rural and urban Bangladesh. Health Science Reports. 2024;7(2):e1849.
19. Disu TR, Anne NJ, Griffiths MD, Mamun MA. Risk factors of geriatric depression among elderly Bangladeshi people: a pilot interview study. Asian journal of psychiatry. 2019;44:163-9.

Figure	[Click here to access/download;Figure;Figure 1.tif](https://www2.cloud.editorialmanager.com/pgph/download.aspx?id=507287&guid=21760017-ee5b-4c84-974a-a5852cb1a13f&scheme=1)[<https://www2.cloud.editorialmanager.com/pgph/download.aspx?id=507287&guid=21760017-ee5b-4c84-974a-a5852cb1a13f&scheme=1>](https://www2.cloud.editorialmanager.com/pgph/download.aspx?id=507287&guid=21760017-ee5b-4c84-974a-a5852cb1a13f&scheme=1)

Figure	[Click here to access/download;Figure;Figure 2.tif](https://www2.cloud.editorialmanager.com/pgph/download.aspx?id=507288&guid=0d04f62f-ffe7-4da1-a008-bdd5f5668488&scheme=1)[<https://www2.cloud.editorialmanager.com/pgph/download.aspx?id=507288&guid=0d04f62f-ffe7-4da1-a008-bdd5f5668488&scheme=1>](https://www2.cloud.editorialmanager.com/pgph/download.aspx?id=507288&guid=0d04f62f-ffe7-4da1-a008-bdd5f5668488&scheme=1)

GDS-SF License Agreement

[Click here to access/download](https://www2.cloud.editorialmanager.com/pgph/download.aspx?id=517591&guid=89933d64-568a-41f2-a391-9bf7ceb02fba&scheme=1)

[Supporting Information renamed_e7b10.pdf](https://www2.cloud.editorialmanager.com/pgph/download.aspx?id=517591&guid=89933d64-568a-41f2-a391-9bf7ceb02fba&scheme=1)

GDS-SF translated questionnaire

[Click here to access/download](https://www2.cloud.editorialmanager.com/pgph/download.aspx?id=518236&guid=4633dddd-c45e-4244-b80c-03d94f426dc5&scheme=1)

[Supporting Information](https://www2.cloud.editorialmanager.com/pgph/download.aspx?id=518236&guid=4633dddd-c45e-4244-b80c-03d94f426dc5&scheme=1)

[GDS Bangla translated questionnaire 250325.docx](https://www2.cloud.editorialmanager.com/pgph/download.aspx?id=518236&guid=4633dddd-c45e-4244-b80c-03d94f426dc5&scheme=1)
