# Supplementary material for: Translation, cultural adaptation, and validation of the Geriatric Depression Scale – Short Form into Bengali among elderly people in Bangladesh
Source: PLOS Glob Public Health. 2026 Mar 19;6(3):e0005341. doi: 10.1371/journal.pgph.0005341 (PMC13001932; doi:10.1371/journal.pgph.0005341)
Supplement: Attachment — Submitted filename: PGPH-D-25-00588_revisionskrl.docx [file pgph.0005341.s003.docx]

This paper is on an important topic and the introduction justifies the research question well. However, there are substantial and significant revisions needed to clarify the methodology and results before it is suitable for publication. Without substantial revision and clarification, I cannot recommend

Introduction

Page 4-5: What are unique challenges to adapting the tool for this context? Have any other tools been previously adapted and what if anything is currently used? It would be helpful to know more background on current standard of care (even if only a few sentences).

Methods

*Study Design and Study setting*

This section needs more information on who participants are (including why they were seen at this hospital/study site) and the context of the study site. Additional typo edits.

What were the inclusion and exclusion criteria? Please clarify, including the age limits if any.

It is unclear where participants were recruited from. Based on the first paragraph, it appears they would be recruited from one study site. However, this is contradicted later on in the section. Please clarify, including why participants were seeking medical care, where they were recruited, and the type of sampling used.

*Translation and Cultural Adaptation*

The process of cultural adaptation needs to be described. Was the tool pilot tested? If so, among who and how many people? What was the process for adapting? This is critical to the methods and results of the paper.

“Participants were aged 60 years or older and were recruited from diverse community settings to ensure variability in socio-demographic and health-related characteristics.”

What type of recruitment? This needs to be clarified.

How did you define “diverse” and ensure representation”

“The sample size was determined to provide sufficient statistical power to detect meaningful associations across depression categories (no depression, mild depression, and moderate depression).”

Please report the sample size calculation.

“Efforts were made to include individuals with varying levels of education, marital status, family types, and comorbid conditions to ensure the generalizability.”

What efforts? This needs clarification.

*Statistical analysis*

Provide citation for statistical software.

What type of chi-square tests were used?

What type of correlation analyses were used?

Please describe the application of PCA more thoroughly.

Results

Grammatical edits included in paper.

“The socio-demographic characteristics are detailed out in Table 1.” Please summarize and clarify in paper.

Table 1. Please indicate what tests were used for which p-values. It is unclear what tests were used for which variables and tables.

“Among participants with mild depression, 61.7% were ex- or current smokers, while 38.3% were non-smokers. Similarly, 52.9% of participants with moderate depression were ex- or current smokers.” Can remove.

The methodology for calculating sensitivity and specificity is unclear, This needs to be clarified in the methods section as does the gold standard measurement used to compare the adapted GDS-15. What were the PPV and NPV?

Again, it is unclear what cultural adaptation methods were used and how this ensured culturally appropriate adaptation.

Discussion/conclusions

Claims of diagnostic accuracy are currently not supported as it is unclear how this was measured.

Be cautious with the word ‘reveal’. Support vs. do not support would be better language here.

“A key strength of this study was the rigorous translation and cultural adaptation process,” Again, this process is unclear. While it might have been rigorous, there is no evidence of this for cultural adaptation processes.
