# Supplementary material for: Translation, cultural adaptation, and validation of the Geriatric Depression Scale – Short Form into Bengali among elderly people in Bangladesh
Source: PLOS Glob Public Health. 2026 Mar 19;6(3):e0005341. doi: 10.1371/journal.pgph.0005341 (PMC13001932; doi:10.1371/journal.pgph.0005341)
Supplement: Attachment — Submitted filename: Response to reviewers.docx [file pgph.0005341.s004.docx]

**Response to reviewers’ comments:**

Manuscript Title: Translation, Cultural Adaptation, and Validation of the Geriatric Depression Scale – Short Form into Bengali among Bangladeshi Elderly People

Manuscript Number: PGPH-D-25-00588

We thank the reviewers for their thoughtful and constructive feedback, which has substantially improved the clarity and rigor of our manuscript. Below, we provide a detailed point-by-point response to each comment, with references to changes made in the revised manuscript (tracked version).

1. **Reviewer’s comments:**

*This paper is on an important topic and the introduction justifies the research question well. However, there are substantial and significant revisions needed to clarify the methodology and results before it is suitable for publication. Without substantial revision and clarification, I cannot recommend*

**Response:**

We have significantly revised the Methods and Results sections to improve clarity, expand methodological detail (particularly regarding participant recruitment, cultural adaptation, and sensitivity), and ensured all statistical procedures are fully described. Specific responses to each methodological concern are outlined below.

2. **Reviewer’s comments:**

*Introduction*

*Page 4-5: What are unique challenges to adapting the tool for this context? Have any other tools been previously adapted and what if anything is currently used? It would be helpful to know more background on current standard of care (even if only a few sentences).*

**Response:**

We have expanded the Introduction to include a paragraph discussing the unique challenges of adapting mental health screening tools in Bangladesh, such as literacy barriers, mental health stigma, and the predominance of informal care practices. We also referenced existing adaptations of tools and clarified the lack of standardized geriatric depression screening in current practice.

3. **Reviewer’s comments:**

*Methods*

*Study Design and Study setting*

*This section needs more information on who participants are (including why they were seen at this hospital/study site) and the context of the study site. Additional typo edits.*

*What were the inclusion and exclusion criteria? Please clarify, including the age limits if any.*

**Response:**

We clarified that participants were purposively sampled from the geriatric ward of Chittagong Medical College Hospital (CMCH), a tertiary care institution. We added details about the context of the hospital, the reason for participant admissions, and the demographics typically served. Inclusion criteria (age ≥60, ability to provide consent) and exclusion criteria (severe cognitive impairment or acute psychiatric illness) have now been explicitly stated.

4. **Reviewer’s comments:**

*It is unclear where participants were recruited from. Based on the first paragraph, it appears they would be recruited from one study site. However, this is contradicted later on in the section. Please clarify, including why participants were seeking medical care, where they were recruited, and the type of sampling used.*

**Response:**

The section has been revisited

5. **Reviewer’s comments:**

*Translation and Cultural Adaptation*

*The process of cultural adaptation needs to be described. Was the tool pilot tested? If so, among who and how many people? What was the process for adapting? This is critical to the methods and results of the paper.*

**Response:**

A section has been added in the method section for cultural adaptation

6. **Reviewer’s comments:**

*“Participants were aged 60 years or older and were recruited from diverse community settings to ensure variability in socio-demographic and health-related characteristics.”*

*What type of recruitment? This needs to be clarified.*

*How did you define “diverse” and ensure representation”*

**Response:**

We clarified that purposive sampling was used to ensure diversity in education level, marital status, family type, and comorbidity profile. We now define “diverse” explicitly and describe how these characteristics were balanced in the sample.

7. **Reviewer’s comments:**

*“The sample size was determined to provide sufficient statistical power to detect meaningful associations across depression categories (no depression, mild depression, and moderate depression).”*

*Please report the sample size calculation.*

**Response:**

We have reported the sample size calculation in the main manuscript.

8. **Reviewer’s comments:**

*“Efforts were made to include individuals with varying levels of education, marital status, family types, and comorbid conditions to ensure the generalizability.”*

*What efforts? This needs clarification.*

**Response:**

This sentence is removed from the manuscript

9. **Reviewer’s comments:**

*Statistical analysis*

*Provide citation for statistical software.*

**Response:**

Citation for Stata was provided

10. **Reviewer’s comments:**

*What type of chi-square tests were used?*

**Response:**

We used Pearson's chi-square for categorical comparisons

11. **Reviewer’s comments:**

*What type of correlation analyses were used?*

**Response:**

We used Pearson's correlation test

12. **Reviewer’s comments:**

*Please describe the application of PCA more thoroughly*

**Response:**

We expanded our description of PCA, explaining the extraction criteria (eigenvalues >1), use of varimax rotation, and interpretation of variance explained and component structure.

13. **Reviewer’s comments:**

*Results*

*Grammatical edits included in paper.*

*“The socio-demographic characteristics are detailed out in Table 1.” Please summarize and clarify in paper.*

*Table 1. Please indicate what tests were used for which p-values. It is unclear what tests were used for which variables and tables.*

**Response:**

We revised the narrative to provide a concise summary of key demographic characteristics and removed the redundant details about smoking. Statistical test types are now clearly indicated in table footnotes.

14. **Reviewer’s comments:**

*“Among participants with mild depression, 61.7% were ex- or current smokers, while 38.3% were non-smokers. Similarly, 52.9% of participants with moderate depression were ex- or current smokers.” Can remove.*

**Response:**

Revised

15. **Reviewer’s comments:**

*The methodology for calculating sensitivity and specificity is unclear, This needs to be clarified in the methods section as does the gold standard measurement used to compare the adapted GDS-15. What were the PPV and NPV?*

**Response:**

In the Data analysis section, a description was added, and also, PPV and NPV are reported in the paper

16. **Reviewer’s comments:**

*Again, it is unclear what cultural adaptation methods were used and how this ensured culturally appropriate adaptation.*

**Response:**

Cultural adaptation has been detailed out in the method section

17. **Reviewer’s comments:**

*Discussion/conclusions*

*Claims of diagnostic accuracy are currently not supported as it is unclear how this was measured.*

*Be cautious with the word ‘reveal’. Support vs. do not support would be better language here.*

*“A key strength of this study was the rigorous translation and cultural adaptation process,” Again, this process is unclear. While it might have been rigorous, there is no evidence of this for cultural adaptation processes.*

**Response:**

We revised phrases like “reveal” to “support” and replaced overly definitive language. We emphasized that the findings suggest the tool’s promise but also acknowledged limitations such as low internal consistency. We now support this claim with detailed description of the adaptation process in the Methods and reaffirm it in the Discussion. We cite methodological references (Borsa et al., 2012; Arafat et al., 2016) to reinforce the rigor of our process.
