# Supplementary material for: Translation, cultural adaptation, and validation of the Geriatric Depression Scale – Short Form into Bengali among elderly people in Bangladesh
Source: PLOS Glob Public Health. 2026 Mar 19;6(3):e0005341. doi: 10.1371/journal.pgph.0005341 (PMC13001932; doi:10.1371/journal.pgph.0005341)
Supplement: Attachment — Submitted filename: Rebuttal_Letter_2_PGPH_D_25_00588 011025.docx [file pgph.0005341.s005.docx]

# Response to Reviewers

**Manuscript ID: PGPH-D-25-00588**

**Title:** Translation, Cultural Adaptation, and Validation of the Geriatric Depression Scale – Short Form into Bengali among Bangladeshi Elderly People

Dear Editor and Reviewers,

We thank the reviewers and editorial team for their constructive feedback on our manuscript. We have carefully revised the paper in line with the comments. Below, we provide a detailed, point-by-point response to each reviewer’s suggestions, indicating the changes made. All revisions are highlighted in the tracked version of the manuscript.

**Reviewer #1**

**Comment 1:** Concern about representativeness, as all subjects were from geriatric wards, possibly with chronic diseases.

*Response:* We clarified in the Study Population section that although participants were recruited from a geriatric unit, they came from diverse communities with a variety of comorbid conditions and were not exclusively chronic disease patients.

**Comment 2:** Exclusion criteria may have overlooked other conditions (severe physical diseases, language disorders, etc.).

*Response:* We added details specifying that inclusion required adequate communication ability, and that individuals with severe physical illnesses or language disorders impairing participation were not enrolled.

**Comment 3:** Pre-test with only 5 elderly participants may not have fully captured cultural adaptability.

*Response:* We acknowledged this limitation in the Strengths and Limitations section and noted that while 5 participants were included in line with recommended pilot practices, future work should involve larger pilot samples.

**Reviewer #3**

**General Comment:** Interesting study, but grammar, referencing, and clarity need improvement.

*Response:* We have corrected grammatical errors and ensured consistent APA-style reference throughout the manuscript.

**Comment 1 (Title):** Suggested revised title.

*Response:* The title has been updated to: “Translation into Bengali, Cultural Adaptation, and Validation of the Short Form Geriatric Depression Scale among Elderly People in Bangladesh.”

**Comment 2 (Abstract):** Clarify the meaning of “Bengali” and ensure conclusions reflect findings.

*Response:* We clarified that “Bengali” refers to the Bangla language and revised the abstract’s conclusion to directly reflect study findings (validity demonstrated but reliability low, requiring refinement).

**Comment 3 (Introduction):** Knowledge gap on geriatric depression assessment in Bangladesh unclear.

*Response:* We expanded the Introduction to emphasize the absence of standardized geriatric depression screening tools in Bangladesh and the need for validated instruments.

**Comment 4 (Methods):** Use “Methodology” instead of “Materials and Methods,” expand details of study setting, sampling, and translation process.

*Response:* Section renamed to “Methodology.” Additional details added on setting, purposive sampling, inclusion/exclusion criteria, and the forward–backward translation with expert review.

**Comment 5 (Results, Tables):** Revise tables for clarity and consistency (% (n) format, units, labeling).

*Response:* All tables revised to present results with notation where appropriate

**Comment 6 (Discussion/References):** Ensure referencing style uniformity.

*Response:* Revisited and corrected

**Comment 7 (Conclusion)**: Conclusion should reflect study findings.

Response: Revised conclusion to state that the tool is valid but requires refinement for reliability before routine use.

**Reviewer #4**

**Comment:** Content validation missing; CVI table recommended.

*Response:* We have added a Content Validity subsection under Methodology. A panel of bilingual geriatric experts rated each item for relevance, clarity, and cultural appropriateness using a 4-point Likert scale. The calculated Content Validity Index (CVI) ranged from 0.80 to 1.00, indicating excellent agreement. A new table has been added to the manuscript as a supplementary material

**Additional Revisions**

- Language polished for clarity and conciseness.

- Uniform APA-style referencing

- Figures reformatted for consistency.

- Strengths and Limitations section expanded to acknowledge small pilot size, single-center sampling, and low internal consistency.

We sincerely thank the reviewers for their insightful comments, which have significantly improved the quality and clarity of our manuscript. We hope that the revised version addresses all concerns and will now be considered suitable for publication in PLOS Global Public Health.

Sincerely,

Mahmud Hassan Arif (Corresponding Author)

On behalf of all co-authors
