# Supplementary material for: Translation, cultural adaptation, and validation of the Geriatric Depression Scale – Short Form into Bengali among elderly people in Bangladesh
Source: PLOS Glob Public Health. 2026 Mar 19;6(3):e0005341. doi: 10.1371/journal.pgph.0005341 (PMC13001932; doi:10.1371/journal.pgph.0005341)
Supplement: Attachment — Submitted filename: Response to the reviewers_V3.docx [file pgph.0005341.s008.docx]

**Response to the reviewers’ comments for the manuscript PGPH-D-25-00588R2**

**Reviewer #3:**

**Comment:**

The authors have tried to address the comments raised. However, the authors have not put attention to all the comments that were suggested i.e. Title: “Translation into Bengali language, Cultural Adaptation, and Validation of the short form Geriatric Depression Scale among Elderly People in Bangladeshi” was suggested but was not effected.

***Response:*** *Thank you for your kind suggestion. We have revised the title accordingly*.

**Comment:**

Abstract
The authors need to draw the conclusion based on the findings, which is not the case in the present paper. This was not affected as well.

***Response:*** *We would like to thank the reviewer for keen observation. We have revisited the conclusion in the abstract section.*

**Comment:**

Results
The table 1 needs to be revised to make it clear and the authors should explain what the values in the brackets mean. Do the authors mean n(%) in table 1? Suggestion, the subheading in the first row for the different column should include % (n). Otherwise, these values do not make sense. In table 3 and 4, the authors have not responded to the following comments. The values should be presented as % (n).

***Response:*** *We have included the notations %(n) in Tables 1, 2, and 3*

**Reviewer #4:**

**Comment:**

The authors have completed some essential processes needed to validate their tool like PCA and reliability. There are a few steps that could have been included but the processes completed are enough to determine the validation.

***Response:*** *Thank you very much for your knowledgeable feedback on this paper*
